# The Role of Prosocialness and Trust in the Consumption of Water as a Limited Resource

**DOI:** 10.3389/fpsyg.2017.00694

**Published:** 2017-05-08

**Authors:** Esther Cuadrado, Carmen Tabernero, Rocío García, Bárbara Luque, Jan Seibert

**Affiliations:** ^1^Instituto Maimónides de Investigacion Biomédica de CórdobaCórdoba, Spain; ^2^Department of Psychology, University of CórdobaCórdoba, Spain; ^3^Department of Social Psychology, University of SalamancaSalamanca, Spain; ^4^Department of Geography, University of ZurichZurich, Switzerland

**Keywords:** water, simulation, competition/cooperation, mediation/moderation, prosocialness, trust

## Abstract

This research analyzes the role of prosocialness and trust in the use of water as a limited resource under situations of competition or cooperation. For this purpose, 107 participants played the role of farmers and made decisions about irrigating their fields in the web-based multiplayer game Irrigania. Before the simulation exercise, participants’ prosocialness and trust levels were evaluated and they were randomly assigned to an experimental condition (competition or cooperation). Repeated measures analysis, using the 10 fields and the experimental conditions as factors, showed that, in the cooperation condition, farmers and their villages used a less selfish strategy to cultivate their fields, which produced greater benefits. Under competition, benefits to farmers and their villages were reduced over time. Mediational analysis shows that the selfish irrigation strategy fully mediated the relationship between prosocialness and accumulated profits; prosocial individuals choose less selfish irrigation strategies and, in turn, accumulated more benefit. Moreover, moderation analysis shows that trust moderated the link between prosocialness and water use strategy by strengthening the negative effect of prosocialness on selection of selfish strategies. The implications of these results highlight the importance of promoting the necessary trust to develop prosocial strategies in collectives; therefore, the efficacy of interventions, such as the creation of cooperative educational contexts or organization of collective actions with groups affected by water scarcity, are discussed.

## The Importance of Studying Water Consumption

Water considered as the blue gold of the 21st century (Barlow and Clarke, 2005) is an increasingly scarce natural resource (Barker et al., 2012). As Katz (2011) claims “*Water has been and continues to be a source of political conflict, at times even violent conflict, a prospect which may worsen as populations grow, economies develop, and climatic conditions change*” (p. 29). Water is an essential to human life common pool natural resource; therefore, it is a scarce and precious resource, particularly in places where there is a great shortage of water, mainly due to the climate, leading to serious problems for society and for the environment. Therefore, water scarcity and interdependent collective behavior in which individuals tend to maximize their personal benefit create the necessary conditions for considering water consumption as a social dilemma. In this sense, for example, applied research has explored the positive impact of interventions in real-world dilemmas related to water conservation by publishing data on private consumption (Van Vugt and Samuelson, 1999).

The way society uses common-pool natural resources is an issue of great contemporary relevance. Gifford (2014) claims that influences on pro-environmental behavior interact, moderate, and mediate each other to predict certain behavioral outcomes. It therefore seems relevant to examine the behavior associated with water consumption from both an individual and a collective perspective, and to analyze how certain variables influence pro-environmental behavior. To investigate the factors and processes underlying water use strategies, this study used a simulation in which the participants had to make decisions about how to use water a precious and scarce resource under different experimental conditions: either cooperation or competition. In short, this research aimed to analyze the role of some dispositional variables and a situational variable, i.e., competition vs. cooperation, in the use of water as a limited resource.

### Social Dilemmas: Competition and Cooperation in the Use of Water

For decades, several fields, including biology, psychology, education, and economics, have investigated social dilemmas (for a review see Dawes, 1980; Kollock, 1998; Balliet, 2010). Following with the essential idea formulated by Hardin (1968), social dilemmas are situations in which a conflict exists between maximizing one’s individual benefit and maximizing the benefit and wellbeing of the collective (Feeny et al., 1990; Zhong et al., 2013). The implicit temporal dimension in social dilemmas affects both individuals and the collective: consequences for both the individual and the collective can be short- or long-term (Van Lange et al., 2013). Recently Van Lange et al. (2013) theorized that a wide variety of psychological factors play a part in determining behavior in social dilemmas (e.g., social values and trust), and argued that social dilemmas should be analyzed as dynamic interactions between individual’s dispositional characteristics (such as prosocialness or trust levels) and the group’s cooperative situations over time. Following Sagiv et al. (2011), who claimed that fewer studies have investigated how individual differences (e.g., social motives) impact on cooperation and competition, we suggest that the impact of prosocialness and trust as factors that could trigger cooperation in social dilemmas has not been addressed thus far.

According to Zhong et al. (2013, p. 2), “*Social dilemmas describe conflict situations existing between a rational individual maximizing its own benefit and a social group pursuing collective wellbeing*.” In other words, individuals should make a choice by prioritizing either the personal or the collective interest (Parks et al., 2013). Sometimes, the apparently rational selfishness of the individual leads to the collective becoming worse off and, thus, the apparently rational individual also suffers from the problems they imposed on the collective (Kollock, 1998).

From a social dilemma perspective, the decline of common-pool natural resources happens because individuals try to maximize their own short-term interests, regardless of the long-term repercussions of their selfish decisions for the community and the planet (Joireman et al., 2009). Steg and Vlek (2009) pointed out that individuals usually justified their decisions about environment-related behaviors in terms of choosing the alternative that will provide the highest benefit at the lowest cost. Nevertheless, in many cases, behavior is not planned or reasoned, but automatic, following unconscious cognitive patterns (Steg and Vlek, 2009). Research has shown that a prosocial disposition makes an individual think and behave in a more collective way, using more cooperative strategies; contrariwise, selfish individuals tend to behave more individualistically, being more competitive and using more selfish strategies (De Cremer and Van Lange, 2001).

Following with the relevance of cooperation, Aumann and Schelling (2005) claimed that cooperative behavior is primordial and necessary to create a prosperous society, particularly in situations of interdependence, as is the case when sharing common-pool natural resources, where, owing to their scarcity and exhaustibility, the situation also includes some uncertainty, perceived vulnerability, and risk. Nevertheless, in order to cooperate, individuals need to lay aside their self-interest to protect the interests of others (Tomasello and Vaish, 2013). It is also well known that interdependence situations that involve uncertainty and risk may lead individuals to attempt to maximize personal benefit, regardless of the potential negative consequences of this behavior for the collective to which they belong; sharing a limited number of resources makes this behavioral strategy more likely (Tabernero et al., 2014; Balliet et al., 2016). Although individuals know that cooperation might be more beneficial for the community, there is still a tendency to act self-sufficiently (Weber et al., 2004). On the other hand, Kollock (1998) argued that communication increases the frequency of cooperation rates, and several studies have shown that cooperation increases significantly when individuals are given the chance to talk with each other and make public commitments (Lokhorst et al., 2009; Balliet, 2010).

However, it has also been shown that individuals tend to use more selfish strategies and competitive behaviors when in a competitive situation, while in cooperative contexts they tend to be more prosocial (Reeson and Tisdell, 2010). Moreover, in a competitive situation, the prosocial tendencies of individuals are not maintained, and they show self-interested profit behaviors (Reeson and Tisdell, 2010). Similarly, social dilemma research has shown that, when resources are scarce, individuals’ behavior becomes more selfish (Gifford, 2011b; Van Lange et al., 2013). In this sense, Barker et al. (2012) have demonstrated that when in-groups are exposed to competitive contexts their cooperation and profits are reduced.

Based on this research, we predicted that, under competitive conditions, both individuals and groups would generate lower incomes than under cooperative conditions. We also predicted that, in a competitive situation, both individuals and groups would tend to adopt selfish strategies, while, in a cooperative situation, both individuals and groups would tend to use less selfish strategies.

Drawing on traditional social psychology studies (Sherif, 1936), which stated that individuals need frameworks on which to base their actions, Tabernero et al. (2007) showed that the previous experience of a group is a fundamental factor in the future decisions of both individual members and the group as a whole. The competitive or cooperative culture of the group influences how competitive or cooperative the individual and the group will be in the future (Tabernero et al., 2007). It has been argued that, in a competitive situation, the profits of the individuals and the group will be reduced (Barker et al., 2012), as a result, it is predicted that both individuals and groups will see their net profits reduce over time when in a group with a competitive culture, but they will see their net profits increase over time when in a group with a cooperative culture.

H1. Individuals will earn higher net and accumulated incomes in the cooperation condition than the competition condition.
H2. Individuals in the competition condition will see their net profits reduce over time, whilst individuals in the cooperation condition will see their net profits increase over time.
H3. In the competition condition, individuals will use a selfish strategy, while, in the cooperation condition, both individuals and villages will use more prosocial irrigation strategies.

### Psychosocial Variables Related with Pro-environmental Behavior

As it is formulated in the Cognitive Affective Personality System (CAPS; Mendoza-Denton et al., 2001), situations and dispositional characteristics of individuals interact with each other and, consequently, influence individual behavior. As such, in this section, we will focus on the importance of specific psychosocial variables that may affect individual pro-environmental behavior.

The more that individuals perceive environmental threats to their wellbeing, such as water scarcity, the more likely they are to engage in environmental practices, such as water conservation (Baldassare and Katz, 1992). In this sense, for example, Niles et al. (2013) recently reported that farmers who are very concerned about the risk of climate change also tend to be more concerned about forthcoming climate-change-related regulations and the plausible impact they may have for the planet. There is, however, a great variability in the concerns that individuals and collectives have about environmental threats. Scientists are therefore interested in the psychological variables related to pro-environmental behaviors and their psychological determinants (Markowitz et al., 2012). According to Cornelissen et al. (2011) and the Social Intuitionist Model’s (Haidt, 2001), an important determinant of the variability in the prosocial behavior of individuals is referred to in terms of social value orientations (SVO) prosocial or proself. SVO are automatic judgments that activate a behavioral pattern and a tendency to either cooperate or to compete. In the same way, Kaiser and Byrka (2011) have shown that environmentalists people with pro-environmental orientation are prone to act in a prosocial way and have a propensity to be more cooperative on behalf of the collective. These studies suggest that individuals differ along a ‘*prosocial propensity dimension*’ (Kaiser and Byrka, 2011), and that this dimension can affect the extent to which an individual’s dispositions and behaviors are prosocial or proself, and competitive or cooperative (Kaiser and Byrka, 2011). Another relevant variable, in terms of pro-environmental behaviors and the changes in such behaviors, is trust (Parks et al., 2013). Mistrust has been conceived as an important barrier to pro-environmental behavioral change (Gifford, 2011a).

Accordingly, in the next to subsection, we will focus on the relation between prosocialness, trust, and pro-environmental behavior.

#### Prosocialness and Pro-environmental Behavior

Lehmann (1999) hypothesized that people with a highly selfish orientation are less prone to behave ecologically. According to De Cremer and Van Lange (2001), there is evidence that people with a prosocial disposition show higher rates of cooperation in a variety of settings. These authors have demonstrated that individuals with a highly prosocial disposition tend to place more importance on cooperation (rather than competition) in social dilemmas, looking for greater opportunities to improve collective results and equality of results or income. As has previously been pointed out, the automatic judgments implicit in social value orientations activate a cooperative behavioral pattern (Cornelissen et al., 2011), showing that the ‘*prosocial propensity dimension*’ along which individuals differ affects the extent to which an individual’s behavior will be prosocial or proself (Kaiser and Byrka, 2011).

In accordance with these results, it is predicted that individuals with a highly prosocial disposition will adopt less selfish water irrigation strategies, regardless of whether they are in a cooperation or competition situation. In view of our earlier argumentation that when individuals adopt more competitive, more selfish strategies their profits are reduced (Barker et al., 2012), it is predicted that water irrigation strategies will mediate the relation between prosocial disposition and profits.

H4. Controlling the effect of experimental conditions (cooperation vs. competition), selfish strategies will mediate the relation between prosocialness and accumulated income, such that individuals with the most strongly prosocial disposition will use less selfish strategies, which will result in higher accumulated income.

#### Trust and Pro-environmental Behaviors

Trust is fundamental to healthy relationships based on cooperation and unselfish relational strategies. As Twenge et al. (2007) argued, trust is a *sine qua non* of cooperation. When individuals mistrust the goodwill of another, they tend not to engage in prosocial behaviors or provide help. Trust is a dispositional variable that affects the decision to cooperate or defect in social dilemmas (Parks et al., 2013). Like dispositional prosocialness, trust predisposes individuals to act prosocially. It has been shown that individuals who place high levels of trust in others tend to use less selfish strategies and engage in more cooperative behaviors (Parks et al., 1996). Gupta and Ogden (2009) have shown that ‘high thrusters’ engage in more pro-environmental actions than ‘low thrusters’.

With regard to common-pool natural resources and pro-environmental behaviors, it must be recognized that trust may have a special relevance in situations of perceived vulnerability, such as when individuals are sharing a limited, scarce, and precious natural resource, such as water. When individuals assess a situation as risky and believe that their resources are in danger and could be exploited by others (McCarter et al., 2011), i.e., when they feel very vulnerable, their trust levels must be high if they are to behave prosocially and engage in cooperative pro-environmental behaviors.

Parks et al. (2013) argued that trust moderates the effect of social values orientations (such as prosocial disposition) on cooperation, and that prosocial individuals tend to use less selfish strategies and to be more cooperative. We can, thus, hypothesize that, when prosocial individuals place high trust in the prosocial propensity of others, they will tend to be even more cooperative and to use even less selfish strategies than when they do not trust in the prosocial propensity of others. Contrariwise, when proself individuals lack trust in the prosocial propensity of others, they will tend to be even more selfish in their use of strategies than when they trust in the prosocial propensity of others. We predicted that trust would moderate the relationship between prosocialness and water irrigation strategies, regardless of whether individuals are in a situation of competition or cooperation.

H5. Controlling the effect of experimental conditions (cooperation vs. competition), trust will moderate the relation between prosocialness and selfish strategies, such that trust will strengthen the negative association between prosocial disposition and selfish strategies.

## Materials and Methods

### Participants

The participants were 107 students of Environmental Sciences Degrees from the University of Córdoba, 70.5% women and 29.5% men. The mean age was 21.28 years (*SD* = 2.18; range 18–27 years).

### Task and Procedure

#### Task

Irrigania is a game about the shared used of limited water resources, which is played by several interacting participants (Seibert and Vis, 2012; Ewen and Seibert, 2016). The game is implemented as a web-based software and can be played with one computer per student using any web browser. In the game, there are different villages, each comprising a number of farmers. Each farmer (participant) has to maximize net income by deciding how to use their 10 fields each year (the game ran for 10 years). Three irrigation options are available, each with different associated costs and revenues reflecting some aspects of reality. Players could choose between (1) rainfed agriculture: low costs, and low revenue; (2) river water irrigation: high, but fixed cost, revenues high, but reduction if the river water has to be distributed among too many fields in a village; and (3) groundwater-based irrigation: costs increase if the depth of groundwater increases due to overuse, fixed high revenue. Farmers have to choose the number of fields for each of the three options for each round (year). The game is designed so that only a few fields per village can sustainably be irrigated with river water and groundwater, respectively. If more fields are irrigated in one of these two ways, the revenue is reduced (river water) or groundwater levels drop and irrigating with this source of water becomes more expensive up to the point where the costs exceed the income. An important difference between the two ways of irrigation is that there is a memory from year to year for groundwater (carry-over of deficits), whereas, for river water, each year is evaluated independently (no carry-over of deficits). Weather conditions for each year vary randomly between three different states (wet, normal, dry), which changes the number of fields that can be irrigated without overusing resources. The weather conditions also influence the income for the rainfed fields, which increases for wet years and decreases for dry years. Simplifying assumptions in the game are that groundwater and river water are independent and that the different villages do not influence each other. The exact game settings and equations are described and discussed by Seibert and Vis (2012) and Ewen and Seibert (2016).

#### Procedure

Participation in the study contributed toward participants’ degrees, and students participated voluntarily and were informed that the data will be analyzed anonymously and that they can leave the experiment when they wanted. Neither written informed consent nor any ethics committee approval was required before the study began according to the regulations by the Spanish Ministry of Science and Innovation.

Before playing the game, participants completed an on-line survey to provide data on socio-demographic and dispositional variables (first phase). Participants took, on average, 15 min to complete the survey, and their anonymity was guaranteed. In a second phase, participants were randomly assigned to one of two experimental conditions (cooperation or competition) and, finally, in a third phase, participants played the Irrigania game. Participants took between 52 and 84 min to complete the 10 simulated years of the game.

Game information was collected via record sheets that were designed so that each participant could register all of their decisions and achievements. On the record sheet, participants provided information related to the simulation, such as farmer name, village name, village group name, farmer’s decisions (annually: the number of rainfed fields, fields irrigated with groundwater, and fields irrigated with river water), and the outcomes of the simulation (net income per year and accumulated income).

In the first phase of the experiment, participants completed an online questionnaire that was created with the Global Park survey program. They were informed that they would have to do a group task online with other participants. In this phase, and before the group tasks, the socio-demographic and dispositional variables were assessed (prosocialness and trust).

In the second phase, participants were randomly assigned to one of two experimental conditions, cooperation or competition (cooperation *N* = 52; competition *N* = 55). Participants in the cooperation condition could talk to each other with their village’ members about strategies and income whilst they were playing the game (at the end of the years 3, 6, and 9), in order to create a cooperative atmosphere (Kollock, 1998; Lokhorst et al., 2009; Balliet, 2010) in which they can share information about whether strategies were advantageous or not. Moreover, a village-interested objective was introduced to them: they were informed that their main aim in the game was not only to obtain higher individual incomes but also to obtain higher incomes at the village level. In contrast, in the competition condition, no interaction was allowed (at the end of years 3, 6, and 9, the only information available to participants was the recent annual incomes of other village members), and a self-interested objective was introduced to them: they were informed that their main objective was to obtain higher individual incomes. Nineteen villages played in the competition condition, and eighteen villages played in the cooperation condition. In each session, there were approximately 15 farmers, and the majority of villages had 3 farmers (mean = 2.89; min = 2, max = 3).

Finally, in the third phase participants played the Irrigania game (Seibert and Vis, 2012). Once participants completed the game, debriefing information was created and all participants received a seminar about solving social dilemmas associated with scarcity of natural resources.

### Measures

#### Prosocialness

Prosocialness (α = 0.84) was measured using a short version of the Prosocialness Scale (Caprara et al., 2005), which assesses the extent to which individuals engage in sharing, helping, taking care of other’s needs, and empathizing with their feelings. Participants recorded their answers to the 12 items (e.g., ‘I try to console people who are sad’) on a 7-point Likert scale.

#### Trust

Trust (α = 0.87) was measured by adapting the scale used by Greenhalgh and Chapman (1998). The scale has four items and uses a seven-point Likert scale (e.g., ‘I feel that people can be counted on to help me’) that reflect the confidence participants have in the prosocial propensity of others. The scale was presented before the participants were assigned to groups for the online tasks.

#### Analytical Strategies and Decision-Making

In order to measure analytical strategies and decision-making we used the Irrigania simulation game, in which a number of fields are allocated to players, and each player decides how to irrigate each of their fields every year.

##### Selfish irrigation strategy

This was indexed as the difference between the number of fields under groundwater irrigation and the number of fields under rainfed agriculture (groundwater irrigation - rainfed agriculture), using the SPSS program. To generate the “selfish irrigation” measure, we selected groundwater irrigation and rainfed agriculture as the most extreme strategies, selfish and prosocial, respectively, while river water irrigation was not considered for the calculation.

##### Annual and accumulated income

Annual income depended on weather conditions during the previous year, the type of irrigation chosen, and how the other farmers used the water resources. Accumulated income was calculated for years 1 through 10 as the sum of annual income through the 10 years.

### Statistical Analyses

Data were analyzed using SPSS v21. Repeated measure analyses were used to scrutinize the changes over time in the overall sample and in each of the experimental conditions. ANOVAs were performed to explore differences between the cooperation and competition conditions. A mediational analysis was performed by using the Process procedure for SPSS (Hayes, 2013) in order to investigate whether water use strategies mediated the relation between prosocialness and accumulated incomes. A moderation analysis was also performed using Process procedure for SPSS (Hayes, 2013) to corroborate the moderating role of trust in the relation between prosocialness and water use strategies.

## Results

### Effect of the Experimental Condition on Net and Accumulated Incomes (H1)

**FIGURE 1:**
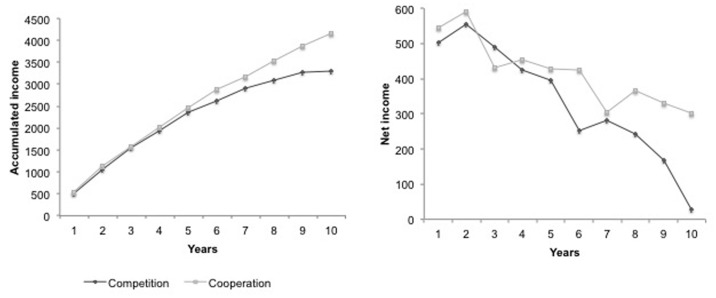
**Accumulated and net income across the 10 years of the simulation at the individual level for both cooperation and competition conditions.** Village level data were almost the same as the individual level data.

The repeated measure analyses (by including the 10 years of accumulated incomes as repeated factor) revealed a significant overall time effect [*F*(9,98) = 459.52, *p* < 0.001; η^2^ = 0.98; *O* = 1.00] over the accumulated incomes throughout the 10 years of the simulation for the complete sample as a whole, as well as a significant time effect for the evolution of accumulated incomes throughout the 10 years in both cooperation [*F*(9,459) = 591.79, *p* < 0.001; η^2^ = 0.99; *O* = 1.00] and competition [*F*(9,46) = 192.80, *p* < 0.001; η^2^ = 0.97; *O* = 1.00] conditions (see **Figure 1**). When including the experimental condition as between-factor (mixed repeated measure analysis), the significant effect of time remain [*F*(9,97) = 461.68, *p* < 0.001, η^2^ = 0.98; *O* = 1.00], a marginal main effect of the experimental condition is observed [*F*(1,105) = 3.09, *p* < 0.09, η^2^ = 0.03; *O* = 0.41], and an interactive effect between time and the experimental condition is observed [*F*(9,97) = 5.94, *p* < 0.001; η^2^ = 0.36; *O* = 1.00].

ANOVAs were used to assess whether the experimental condition (competition vs. cooperation) affected net and accumulated incomes. The results exhibited a significant effect on accumulated income in the 10th year, as well as on the average of net incomes over the 10 years. As expected, individuals in the cooperation competition accumulated higher incomes in the last year of the simulation than individuals in the competition condition [*F*(1,106) = 5.37, *p* < 0.02]. Moreover, the average of net incomes was higher for individuals in the cooperation condition than for individuals in the competition condition [*M*coop = 417.27, *SD* = 56.19; *M*comp = 333.55, *SD* = 272.47; *F*(1,106) = 4.72, *p* < 0.03].

In order to analyze village incomes, a transformation of the matrix was constructed using aggregate measures, for 107 farmers in 37 villages. To support the aggregation, we calculated inter-member reliability (ICC1 and ICC2) and tested whether mean scores differed significantly across groups (indicated by an *F*-test from a one-way ANOVA contrasting group means on each variable). ICC1 indicates the proportion of variance in ratings due to group membership, whereas ICC2 indicates the reliability of group mean differences (Bliese, 2000). Good support for aggregation was obtained for all the net incomes, except for year 1 (**Table 1**); as such, these results demonstrated that the experimental groups actually acted as if consisting of team members who share a common strategy. The results of the analyses at the village level were similar to those at the individual level (see Appendix 1).

**Table 1:** Inter-member reliability and differences in mean scores across groups on the net income variable.

	*F*(36,106)	ICC1	ICC2
Net income yr1	1.25 (ns)	0.08	0.20
Net income yr2	2.95^∗∗∗^	0.40	0.66
Net income yr3	4.36^∗∗∗^	0.54	0.77
Net income yr4	3.17^∗∗∗^	0.43	0.68
Net income yr5	10.56^∗∗∗^	0.77	0.90
Net income yr6	1.64^∗^	0.18	0.39
Net income yr7	6.79^∗∗∗^	0.67	0.85
Net income yr8	1.50^†^	0.15	0.33
Net income yr9	2.30^∗∗∗^	0.31	0.56
Net income yr10	3.10^∗∗∗^	0.42	0.68


*^∗∗∗^*p* < 0.001, ^∗^*p* < 0.05, ^†^*p* < 0.07; ICC, Inter-class correlation.*

These results confirmed that, at both the individual and collective levels, both the net and the accumulated incomes at the end of the simulation were higher for the cooperation condition than for the competition condition (**Figure 1**).

### Evolution of Net Income Over Time According to Experimental Condition (H2)

The repeated measure analyses (including the first and the 10th years of net incomes as factor) of the complete sample as a whole showed significant differences in the evolution of the net income from the first to the 10th years across the two conditions [*F*(1,106) = 62.52, *p* < 0.001, η^2^ = 0.37; *O* = 1.00]. Moreover, as **Figure 1** shows, the repeated measure analyses performed separately with each group showed that, in both the competition [*F*(1,54) = 31.14, *p* < 0.001, η^2^ = 0.37; *O* = 1.00] and cooperation [*F*(1,51) = 169.42, *p* < 0.001, η^2^ = 0.77; *O* = 1.00] conditions, individuals saw their incomes significantly reduce between the first and the 10th year. When doing a mixed ANOVA by including the experimental condition (cooperation vs. competition) as interpersonal factor (between factor), the results showed that the main effect of time remain [*F*(1,105) = 64.65, *p* < 0.001, η^2^ = 0.38; *O* = 1.00], a main effect of the experimental condition is observed [*F*(1,105) = 12.79, *p* < 0.001, η^2^ = 0.11; *O* = 0.94], and a interactive effect is observed showing that the reduction o net income is significantly greater for individuals in the competition condition [*F*(1,105) = 6.67, *p* < 0.01, η^2^ = 0.06; *O* = 0.73]. Hypothesis 2 was therefore partially confirmed.

The results at the village level were similar to those at the individual level, as can be seen in Appendix 2.

### Differences in the Use of Water Strategies According to the Experimental Condition (H3)

**FIGURE 2:**
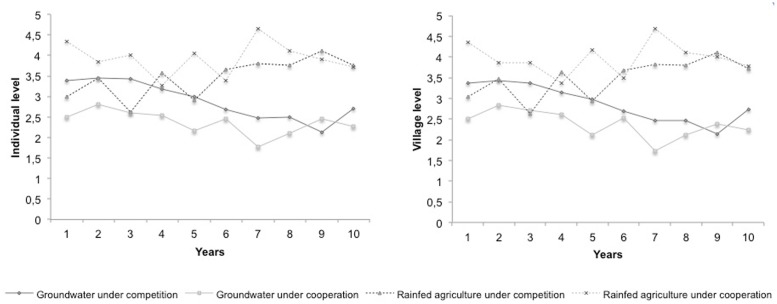
**Number of fields irrigated with groundwater and rainfed agriculture across the simulation**.

We performed ANOVAs to evaluate the irrigation strategies followed by both the cooperation and competition experimental conditions. In the competition condition participants used a more selfish strategy, which resulted in a higher number of fields being irrigated with groundwater (**Figure 2**). On average, over the 10 years, individuals in the competition condition used significantly more groundwater irrigation than individuals in the cooperation condition [*M*comp = 2.89, *SD* = 1.08; *M*coop = 2.37, *SD* = 0.62; *F*(1,106) = 9.25, *p* < 0.01].

Participants in the cooperation condition cultivated a higher number of fields with rainfed agriculture and, thus, used a more prosocial strategy (**Figure 2**). On average, over the 10 years, individuals in the cooperation condition irrigated significantly more fields with rainfed agriculture than individuals in the competition condition [*M*coop = 3.93, *SD* = 0.87; *M*comp = 3.46, *SD* = 1.09; *F*(1,106) = 5.82, *p* < 0.02].

The results at the village level were similar to those at the individual level, as can be seen in Appendix 3.

### Mediating Role of Water Use Strategies on Profits (H4)

In order to test the prediction that water use strategies would mediate the effects of prosocialness on profits, mediation analyses, using Process procedure (Hayes, 2013), were performed; first, without controlling the effect of the experimental condition as covariate; and, then, by controlling the effect of the experimental condition as covariate. At the first step, prosocialness was introduced as independent variable (IV); the mean of selfish irrigation strategy of 5 years previous to the last year of the simulation was introduced as mediator; and the accumulated incomes of the 10th year were introduced as dependent variable (DV). At the second step, in addition to those variables, we also introduced the experimental condition as covariate (coded as -5.00 for competition and 5.00 for cooperation). Since the Process procedure does not accept lost data, these were replaced by the mean of the series. Moreover, for all the quantitative variables, the *z* scores were used. The 95% confidence interval of the indirect effect was obtained with 10,000 bootstrap resamples. As shown in **Table 2**, the results of the analyses revealed that the selfish irrigation strategies significantly mediated the relation between prosocialness and accumulated income. Moreover, when introducing the experimental condition as covariate, the indirect effect of prosocialness on the accumulated incomes remained significant. Hypothesis 4 was thus supported.

**Table 2:** Model coefficients for the mediational process model with and without the experimental condition as control variable.

	Mediational process model without controlling by the experimental condition as covariate								Mediational process model by controlling with the experimental condition as covariate							
		
	Consequent								Consequent							
		
	Selfish irrigation strategy				Accumulated incomes (Y)				Selfish irrigation strategy				Accumulated incomes (Y)			
				
Antecedent		Coeff.	*SE*	*p*		Coeff.	*SE*	*p*		Coeff.	*SE*	*p*		Coeff.	*SE*	*p*
X (Prosocialness)	a	-0.22	0.10	<0.05	c′	0.13	0.10	ns	A	-0.22	0.10	<0.05	c′	0.12	0.09	ns
M (Selfish irrigation strategy)		–	–	–	b	-0.46	0.09	<0.001		–	–	–	b_1_	-0.43	0.09	<0.001
Covariate (Exp. Cond.)			–	–		–	–	–	B_2_	–	–	–	b_2_	0.02	0.02	ns
Intercept	i_1_	-0.01	0.10	ns	i_2_	0.01	0.09	ns	i_1_	-0.01	0.10	ns	i_2_	0.01	0.08	ns
				
	*R*^2^ = 0.04				*R*^2^ = 0.25				*R*^2^ = 0.04				*R*^2^ = 0.26			
	*F*(1,105) = 4.59, *p* < 0.05				*F*(2,104) = 16.89, *p* < 0.001				*F*(1,105) = 4.59, *p* < 0.05				*F*(3,103) = 11.78, *p* < 0.001			

Conditional indirect effect	**95% Bootstrap CI for indirect effect**				**Sobel Test**				**95% Bootstrap CI for indirect effect**				**Sobel Test**			
		
	-0.001 to 0.444				*z*	*SE*		*p*	-0.001 to 0.450				*Z*	*SE*		*P*
					1.95	0.05		<0.05					1.93	0.05		<0.05


*X, independent variable (Prosocialness); M, mediator (Selfish irrigation strategy); Exp. Cond, experimental condition; Y, dependent variable (accumulated incomes).*

### Moderating Role of Trust (H5)

**FIGURE 3:**
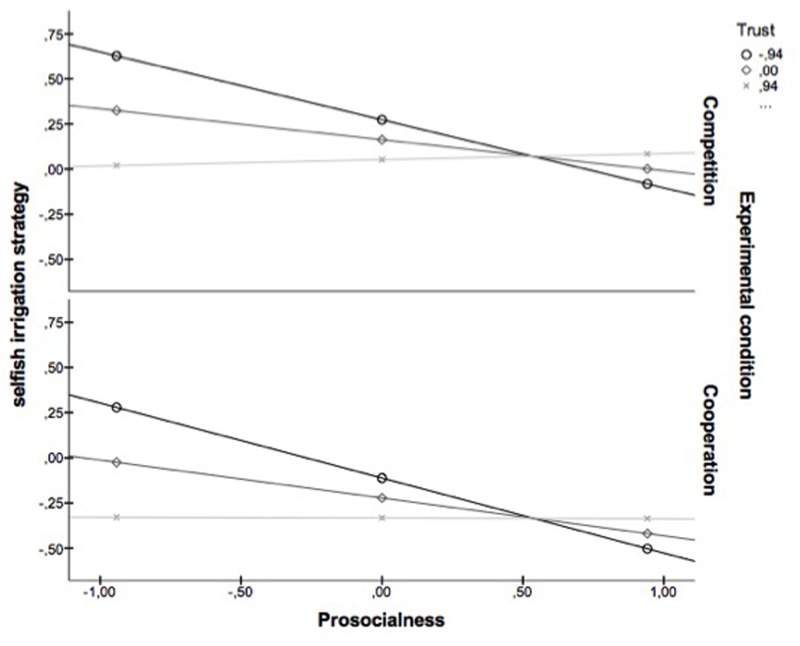
**Moderating role of trust in the link between prosocialness and use of selfish irrigation strategies**.

Moderation analysis was performed using Process procedure in SPSS (Hayes, 2013) in order to confirm Hypothesis 5. Prosocialness was introduced as IV; trust and the experimental condition as moderators; and the selfish irrigation strategies as DV. The experimental condition was introduced as moderator, in order to assess whether or not the possible moderator effect of trust is affected by the experimental condition. Since the Process program does not accept lost data, these were replaced by the mean of the series. Moreover, for all the quantitative variables, the z scores were used. The 95% confidence interval of the indirect effect was obtained with 10,000 bootstrap resamples. As **Table 3** and **Figure 3** show, the results revealed that trust levels strengthen the negative effect that prosocialness had on the selfish irrigation strategies, and that the experimental condition does not moderate the relation between prosocialness and the selfish irrigation strategy. Thus, H5 was confirmed.

**Table 3:** Model coefficients for the moderating effect of trust in the link between prosocialness and selfish irrigation strategies.

		Coeff.	*SE*	*T*	*p*
X (Prosocialness)	b_1_	-0.19	0.10	-1.90	<0.06
M(Trust)	b_2_	-0.12	0.10	-1.15	ns
W (Experimental condition)	b_3_	-0.04	0.02	-2.05	<0.05
XM (Prosocialness × trust)	b_4_	0.22	0.11	2.03	<0.05
XW (Prosocialness × Experimental condition)	b_5_	-0.01	0.02	-0.19	ns
Intercept	i_1_	-0.03	0.09	-0.32	ns
	
	*R*^2^ = 0.13				
	*F*(5,101) = 3.04, *p* < 0.01				

	**Interaction 1 (XM)**		**Interaction 2 (XW)**	**Both**	

*R*^2^ change due to interactions	Δ*R*^2^ = 0.04		Δ*R*^2^ = 0.01	Δ*R*^2^ = 0.04	
	*F*(1,101) = 4.11, *p* < 0.05		*F*(1,101) = 0.03, *ns*	*F*(2,101) = 2.08, *ns*	


*X, independent variable; M, moderator 1; W, moderator 2.*

## Discussion

In our modern and occidental societies competition and maximizing own profit are common frameworks toward resources use; and when framed in competitive situations, the maximization of the own interest increases (Steg and Vlek, 2009). This has obviously negative repercussion for society and the planet. The results of this study seem to indicate that changing this frame by focusing on a more cooperative framework would benefit individual and collective profit as well as resources conservation. The present study showed that, in a simulation, farmers and their respective villages working under competitive conditions earned consistently lower incomes; these results could be explained in terms of the use of more selfish strategies, lower prosocial tendencies, and lower trust levels.

In a cooperative situation, both individuals and groups obtain higher benefits, both net and accumulated, than in a competitive situation, a result in accordance with previous studies (Barker et al., 2012). Moreover, the results of the study also showed that competitive and cooperative situations affect (a) the evolution of the net profits of individuals and groups over time, and (b) the irrigation strategies chosen by both individuals and groups. We found that (a) the net profits of individuals and groups diminished significantly more over time in the competition condition than in the cooperation condition, as Barker et al. (2012) hypothesized; and (b) that individuals in competitive groups tend to choose less pro-environmental and more selfish strategies than individuals and groups in cooperative situations (Reeson and Tisdell, 2010). It is noteworthy that, meanwhile our second hypothesis *Individuals in the competition condition will see their net profits reduce over time, whilst individuals in the cooperation condition will see their net profits increase over time* was not completely confirmed, the reduction of net profits over time was lower when individuals were framed in a cooperation situation. Note that the most prosocial strategy rainfed agriculture used especially by the participants in the cooperation condition, is also the one with the lower revenue (but also the lower costs), and this could have contributed to the not expected reduction of the profits obtained by participants in the cooperation condition. Interestingly, despite the reduction of the incomes over time obtained with the prosocial strategy, participants in the cooperation condition continue to opt for the prosocial strategy; and this decision enhanced finally their benefits to the long-term. Once again, this result strengthens the idea of changing the predominant occidental competitive framework toward sharing resources by promoting cooperative environments for the benefit of individual, societies and the planet.

Furthermore, people usually base their own decisions, in part, on the decisions and behaviors they have observed in others, reciprocation providing one example of this (Joireman et al., 2009). In our study, when participants took part in the simulation, they could see the decisions of other farmers onscreen, and this may have ‘interfered’ with their eventual decision, making it more or less selfish according to the decisions of other participants. Sherif (1936) argued that individuals need to base their actions on frameworks; a cooperative or competitive context might act as a framework and lead individuals to act in accordance with this framework. When a group of people has to share a limited number of resources, there is a tendency to behave selfishly, even if individuals know that mutual cooperation will lead to greater benefits for more people.

Our results seem to corroborate the theories which hold that selfish and self-sufficient behaviors are increased in competitive situations (Weber et al., 2004), and that such self-sufficient behavior has a negative impact in the long-term, not just for the planet and for society in general, when considering natural common-pool resources, but also for the individuals and groups that act self-sufficiently. As Kollock (1998) argued, the apparent rationality of self-sufficient behavior when resources are scarce is not supported by empirical evidence, which has shown that this strategy is detrimental to the individual and the planet. Our results also showed that net benefits for individuals and groups were increasingly and significantly reduced over time in the competitive condition. This suggests that individuals and groups in competitive situations cannot escape the vicious circle in which they are caught and so conflict escalates, becoming more destructive and serious over time. As early as 1990, Deutsch (1990) was claiming that competition tends to escalate into destructive conflict. Nonetheless, our hypothesis that net incomes would increase over time for individuals and groups in the cooperation condition was not confirmed. This is probably due to the fact that, although individuals are in a cooperative context, the goal of the game is competitive and, therefore, the scarcity of resources leads individuals to display some competitive and self-sufficient behaviors (Weber et al., 2004), even in the cooperation condition. Nevertheless, the results showed that, when comparing with competitive situations, the reduction in net income over time in the cooperative condition was significantly lower, both at individual and group level. Cooperation seems to act as a protective factor against the conflict escalation that occurs as a result of the scarcity of resources.

In accordance with Sherif (1936) and Tabernero et al. (2007), the data on the irrigation strategies used in the competition condition more selfish and less pro-environmental and cooperation condition more prosocial and pro-environmental seem to confirm that the competitive or cooperative culture of the in-group influences the behavior of individuals, providing them with a framework on which to base their actions. The more feedback individuals receive about the competitive culture of the group, the more their behavior reflects the competitive context: they act self-sufficiently and make less profit. This suggests that individuals and groups should be provided with some sort of cooperative framework for environment-related decision making, perhaps by creating formative and educational programs that allow individuals to experience (a) cooperation and (b) the benefits that cooperation has at a practical level, both for themselves and for society, as well as for the environment, which ultimately has a further impact on them.

Most authors (Lehmann, 1999; De Cremer and Van Lange, 2001; Cornelissen et al., 2011; Kaiser and Byrka, 2011) agree that prosocial tendencies affect the extent to which individuals behave in a prosocial or proself manner. Our study has shown that individuals with higher prosocialness (a more prosocial disposition) choose more prosocial and pro-environmental irrigation strategies than individuals with lower prosocialness. It seems that dispositional prosocialness can activate a prosocial and pro-environmental pattern of behavior, increasing behaviors that tend to benefit other people and the environment, such as the pro-environmental and prosocial strategies in our simulation. This indicates the importance of educational programs oriented to developing empathy and prosocial dispositions in both children and adults. Our results have also shown that irrigation strategies, in turn, predict incomes, i.e., selfish irrigation strategies fully mediated the relation between prosocialness and profit, such that the more prosocial an individual’s disposition, the less selfish the irrigation strategy they choose and the greater the resultant profit. Once again, this suggests that social and educational programs that can show individuals the benefits for oneself, for society, and for the planet of investment in prosocial strategies and pro-environmental behaviors should be promoted. As Gifford (2014) argues, both knowledge of and education about environmental problems are important factors and agents of change in pro-environmental behaviors. This study and others (Kaiser and Byrka, 2011) have found that, whereas altruistic and prosocial individuals tend to behave in a pro-environmental way, less prosocial individuals tend to pursue self-enhancement through consuming natural resources regardless of the impact such behavior has on other people and the planet; it seems that educational programs would benefit from taking into account the influence of dispositional variables and promoting prosocialness in students and adults.

Given that the results reinforce the thesis focused on the importance of generating confidence for developing less selfish strategies to solve social dilemmas (Van Lange et al., 2013), an important implication of the study focuses on the need to promote group interactions and community meetings over time among groups affected by water scarcity. Previous research has shown (see Ledyard, 1995) that face-to-face meetings prevent uncertainty and vulnerability, as well as facilitating communication, the development of joint strategies, and commitment to the development of collective actions. The individual commitment to contribute to the collective correlates with the expectation of the prosocial behavior of the others (Messick, 1999). In addition, individuals tend to cooperate with those who identify as more prosocial (Feinberg et al., 2014), since the interaction experience can be used to guide how much trust individuals should invest in a given partner (Barclay, 2004). Thus, in future, it would be interesting to investigate if greater prosocialness dispositions can be developed in individuals and collectives by promoting group interactions and community meetings in situations of scarcity of resources.

Finally, the study showed that trust acts as a moderating variable on the relation between prosocialness and selection of selfish strategies, such that trust strengthens the negative effect of dispositional prosocialness on selection of selfish strategies. In other words, prosocialness predicts less selfish strategies, and low trust levels reinforce this relation, whereas high levels of trust do not significantly alter the effect of a prosocial disposition on the selection of selfish strategies. Therefore, proself individuals who lack trust in the prosocialness of others tend to use even more selfish strategies than proself individuals who place high trust in the prosocial disposition of others. The study seems to indicate that, in situations of perceived vulnerability and when dealing with common-pool natural resources, high trust levels are a prerequisite for engaging in cooperative pro-environmental behaviors, especially for individuals with low prosocial tendencies. This is in concordance with previous studies that have shown that trust promotes cooperation (Gupta and Ogden, 2009; Parks et al., 2013), as well as those which argued that trust probably moderates the effect of individual disposition on cooperation (Parks et al., 2013). Trust seems to be a relevant variable when considering practical ways of promoting pro-environmental behavior.

### Limitations

A larger and more heterogeneous particularly with respect to degree subject sample would have been advantageous. It would have been especially beneficial to have a sample of farmers, as they would probably have had more knowledge relevant to the theme of the simulation. In future investigations, it would be interesting to explore other variables that have been shown to have an effect on behavior in social dilemmas, such as self-efficacy. Investigating differences between students of science and arts degrees in terms of previous knowledge of environmental issues and prosocial disposition would also be useful.

## Conclusion

This investigation has explored the role of prosocialness and trust in people’s decision-making with respect to environmental issues specifically the use of water, a limited essential resource and the importance of cooperation in decision-making about common-pool resources. It has shown that there are significant differences between levels of pro-environmental behavior under cooperation and competition conditions. As discussed before, in cooperative conditions the water-use strategies chosen were less selfish and more prosocial and pro-environmental, and this, in turn, affected both net and accumulated income, such that they were higher in the cooperation condition than in the competition condition. This is due to the fact that, in the competition condition, groundwater irrigation was the most frequently used strategy and, while this produces a higher income in the short term, the cost is higher, and thus long-term over-use of this strategy will reduce profits. In the cooperation condition, participants focused more on the common wealth and made more use of rainfed agriculture, thus obtaining a higher income over the long term.

Our study has demonstrated how irrigation strategy mediates the relation between prosocialness and cumulative incomes. Participants with a more prosocial disposition chose less selfish water consumption strategies, and obtained higher cumulative incomes than individuals who used more selfish irrigation strategies. On the other hand, it has shown that trust moderates the relation between prosocialness and selfish irrigation strategies, strengthening the negative association between these two variables. Prosocial individuals with high trust levels behave in an even less selfish way than prosocial individuals with low trust levels.

In conclusion, we would like to reaffirm the relevance, highlighted by Niles et al. (2013), of programs that provide technical assistance aimed at incentivizing voluntary changes in practice through which the agricultural community could achieve environmental benchmarks. Our results suggested that participating in cooperative actions could constitute relevant previous experience for individuals and farmers, promoting development of a cooperative framework that would, in turn, promote more pro-environmental actions at both the individual and collective level, which, consequently, would have benefits for the environment.

## Author Contributions

EC helped to the design of the project, she performed the statistical analyses, and she is the principal redactor of the paper. CT is the main researcher of the project in which the paper is framed, she designed the project, recollected the data with the co-authors, she has collaborated in the redaction of the paper. RG has collaborated in the data recollection and in the redaction of the paper. JS has designed the Irrigania Game used in the experiment, and has collaborated in the redaction of the paper. BL has collaborated in the redaction of the paper.

## Conflict of Interest Statement

The authors declare that the research was conducted in the absence of any commercial or financial relationships that could be construed as a potential conflict of interest.
