# Supplementary material for: The Role of Prosocialness and Trust in the Consumption of Water as a Limited Resource
Source: Front Psychol. 2017 May 8;8:694. doi: 10.3389/fpsyg.2017.00694 (PMC5420575; doi:10.3389/fpsyg.2017.00694)
Supplement: Supplementary file 1 [file Data_Sheet_1.docx]

**Appendix 1. Effect of the experimental condition on net and accumulated incomes (H1) at the village level**

To assess whether there were significant differences between the experimental conditions in terms of accumulated incomes and annual net income, a series of ANOVAs was conducted. Significant differences were found when considering the accumulated incomes over ten years (*F*(1,94) = 5.64 *p* < .02; *M*_coop_ = 4172.75, *SD* = 561.73; *M*_compet_ = 3168.40, *SD* = 2989.21). Significant differences between the experimental conditions were found for the mean of net incomes of the ten years (*F*(1,94) = 4.88, *p* < .03; *M*_coop_ = 417.27, *SD* = 56.19; *M*_compet_ = 322.26, *SD* = 304.15).

**Appendix 2. Evolution of net income over time according to experimental condition (H2) at the village level**

At the village level, the repeated measure analyses of the complete sample as a whole showed significant differences in the evolution of the net income from the first to the tenth years across the two conditions (*F*(1,35) = 36.18, *p* < .001, η^2^ = .51; *O* = 1.00). Moreover, the repeated measure analyses performed separately with each group showed that, in the competition condition, villages saw their incomes significantly reduce between the first and the tenth year (*F*(1,18) = 17.39, *p* < .001, η^2^ = .49; *O* = .98); the reduction was significantly greater for villages in the competition condition (*F*(1,17) = 89.35, *p* < .001, η^2^ = .84; *O* = 1.00). Hypothesis 2 was therefore partially confirmed.

### Appendix 3. Differences in the use of water strategies according to the experimental condition (H3) at the village level

Different ANOVAs were conducted using the aggregated matrix to evaluate the irrigation strategies according to the experimental condition in each village. Villages in the competition condition used a more selfish strategy that led to a higher number of fields being irrigated with groundwater. Results showed that the difference between conditions was significant in the first (*M*comp = 3.42, *SD* = 2.00; *M*coop =2.50, *SD* = 1.26; *F*(1,94) = 7.40; *p* < .01), second (*M*comp = 3.60, *SD* = 2,20; *M*coop = 2.81, *SD* = 1.44; *F*(1,94) = 4.51; *p* < .05), third (*M*comp = 3.42, *SD* = 2.20; *M*coop =, 2.60; *SD* = 1.73; *F*(1,94) = 4.17; *p* < .05), fifth (*M*comp = 3.02, *SD* = 1.83; *M*coop = 2.17, *SD* = 1.23; *F*(1,94) = 7.24; *p* < .01), and seventh (*M*comp = 2.56, *SD* = 1.47; *M*coop = 1.77, *SD* = 1.35; *F*(1,94) = 7.41; *p* < .01) years. Moreover, the mean of fields irrigated with groundwater over the ten years was significantly higher (*F*(1,94) = 10.09, *p* < .01) in the competition condition (*M* = 2.97, *SD* = 1.18) than in the cooperation condition (*M* = 2.37, *SD* = 0.62).

Villages in the cooperation condition used a more prosocial strategy, combining modes of irrigation and using rainfed agriculture (Figure 2). Results showed that the difference between experimental conditions was significant in the first (*M*coop = 4.33, *SD* = 1.98; *M*comp = 2.91, *SD* = 2.17; *F*(1,94) = 11.12; *p* < .001), third (*M*coop = 4.00, *SD* = 2.19; *M*comp = 2.63, *SD* = 1.94; *F*(1,94) = 10.25; *p* < .01), fifth (*M*coop = 4.06, *SD* = 1.90; *M*comp = 3.02, *SD* = 1.87; *F*(1,94) = 7.06 *p* < .01), and seventh (*M*coop = 4.65, *SD* = 2.20; *M*comp = 3.72, *SD* = 2.07; *F*(1,94) = 4.47 *p* < .05) years. Moreover, the mean of fields irrigated with rainfed agriculture over the ten years was significantly higher (*F*(1,94) = 6.42, *p* < .01) in the cooperation condition (*M* = 3.93, *SD* = 0.87) than in the competition condition (*M* = 3.40, *SD* = 1.05).
